# Bacteria into bloodstream caused by oral probiotics based on whole genome sequencing: A case report

**DOI:** 10.1097/MD.0000000000043337

**Published:** 2025-07-11

**Authors:** Tianqi Qi, Yingshi Wang, Yanhui Liu, Wenqiang Li, Shanshan Wu

**Affiliations:** a Department of Clinical Laboratory, Aerospace Center Hospital, Beijing, China; b Department of Gastrointestinal Surgery, Aerospace Center Hospital, Beijing, China; c Department of Gastroenterology, Beijing Friendship Hospital, Capital Medical University, National Clinical Research Center for Digestive Disease, Beijing Digestive Diseases Center, Beijing Key Laboratory for Precancerous Lesions of Digestive Disease, Beijing, China.

**Keywords:** *Bacillus licheniformis*, blood culture, *Lactiplantibacillus plantarum*, probiotics, whole-genome sequencing

## Abstract

**Rationale:**

*Bacillus licheniformis* and *Lactiplantibacillus plantarum* are facultative anaerobes and gram-positive bacteria. They are commonly included in probiotic preparations and are administered orally in clinical practice to promote a balanced gut microbiota.

**Patient concerns:**

An 85-year-old man with irritable bowel syndrome and reflux esophagitis underwent distal pancreatectomy and was administered oral probiotics. Blood culture was positive for *B. licheniformis* and *L. plantarum*. We conducted whole-genome sequencing for homology analysis and pathogenicity prediction of the strains isolated from the patient’s blood culture and oral probiotics.

**Diagnosis:**

The initial diagnosis was bacterial entry into the bloodstream resulting from the consumption of *B. licheniformis* and *L. plantarum* probiotic preparations.

**Interventions:**

The patient was treated with discontinuation of oral probiotics and timely administration of antibiotics.

**Outcomes:**

Follow-up blood culture results after treatment were negative.

**Lessons:**

Probiotics are generally considered relatively safe but should be preceded by risk screening in vulnerable populations. Whole-genome sequencing revealed the potential risks of probiotic use through homology analysis and prediction of virulence factors and antibiotic resistance.

## Graphical abstract

**Figure:**
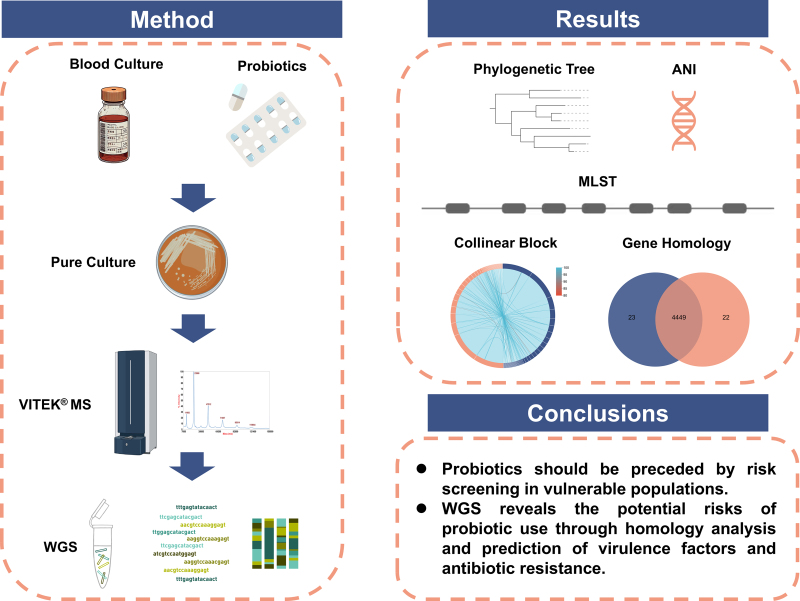

## 1. Introduction

Probiotics are defined as products containing live microorganisms that enhance human or animal health and exert positive effects on tissues and organs.^[1,2]^ The global consumer focus on gut health and immune regulation is driving the rapid growth of the probiotic market. According to 2024 data from Grand View Research and NIH Market Research, the global market size is between $61 billion and $65 billion, with an expected compound annual growth rate of 7 to 8% by 2030. Data from the Web of Science database show that the number of probiotic-related research papers published globally in 2022 increased to 6916, including 5051 studies. The diseases mainly addressed were irritable bowel syndrome, *Clostridium difficile* infection, inflammatory bowel disease, and celiac disease. Many probiotic strains have been classified as “Qualified Presumption of Safety” by the European Food Safety Authority or as “Generally Recognized as Safe” by the US Food and Drug Administration. A unique characteristic of probiotics is that they remain viable upon administration and possess the potential for infectivity or in situ toxin production.^[3]^ Due to the reported infections associated with probiotics, their use should be approached with caution, particularly in vulnerable or at-risk populations.^[4]^ Studies have shown that *Bacillus* spp. can cause bacteremia in extremely low birth weight neonates and in patients with esophageal perforation.^[5,6]^ *Bacillus licheniformis* (*B. licheniformis*) and *Lactiplantibacillus plantarum* (*L. plantarum*) are facultative anaerobic, gram-positive bacteria that are important components of oral probiotic preparations. This case reported both bacterial entry into the blood, probably resulting from the consumption of probiotic preparations, which was proved by whole-genome sequencing (WGS) for homology analysis of bacterial strains.

## 2. Case presentation

An 85-year-old man was admitted to the hospital with “intermittent fever for >2 weeks and pancreatic lesions for 1 week.” He was diagnosed with a pancreatic space-occupying lesion and scheduled for surgery. The patient had a history of Alzheimer disease for >10 years and had severe osteoporosis, irritable bowel syndrome, and reflux esophagitis. He had previously been treated for incomplete intestinal obstruction and was discharged from our hospital. Preoperatively, chest and abdominal CT revealed bilateral ground-glass opacities in both lungs and pancreatic lesions. Physical examination revealed a temperature of 36.8°C, heart rate of 80 bpm, respiratory rate of 18 bpm, and blood pressure of 120/69 mm Hg. The patient exhibited altered consciousness, inability to speak, and coarse bilateral breath sounds. Specialized examination of the abdomen showed no abnormalities, with bowel sounds presented 4 times per minute and a normal anal examination in the chest-knee position.

**Figure 1.**
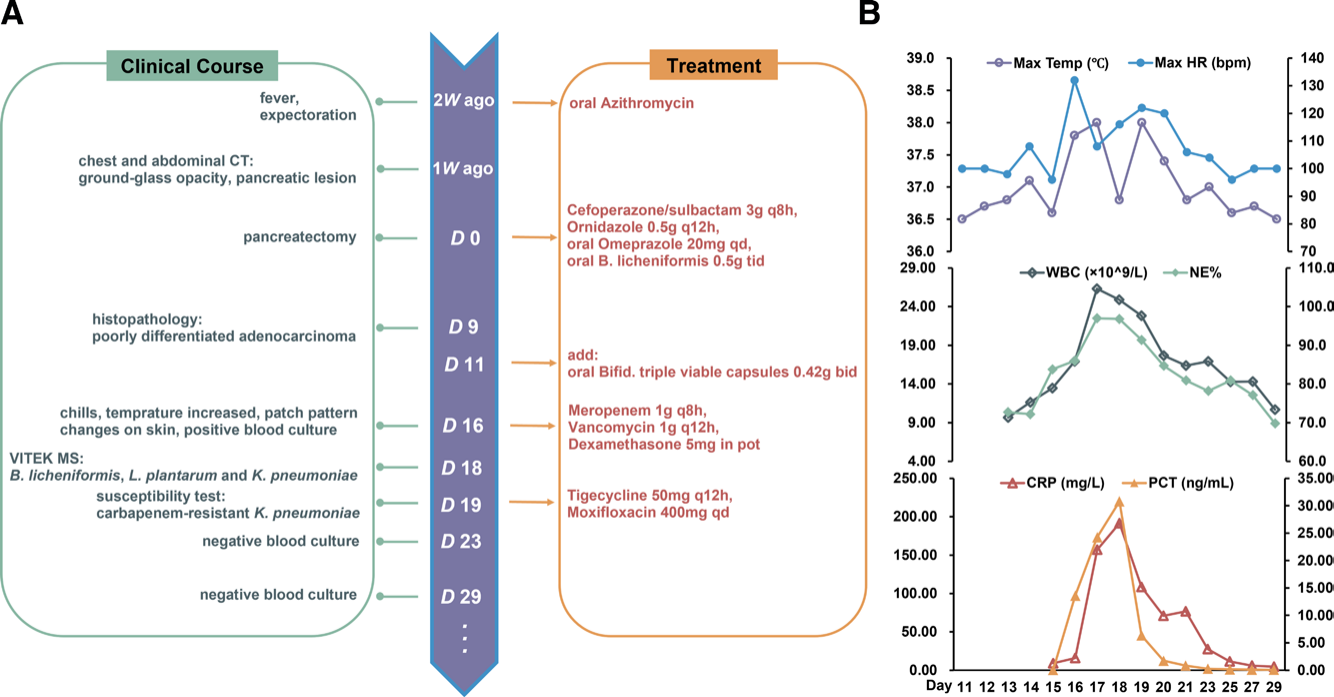
Clinical course, treatment timeline, and laboratory examinations of the patient. (A) Clinical course and antibiotic therapy during patient hospitalization: The green box includes clinical symptoms, imaging, pathology, and microbiological examination, and the orange box includes the drug treatment course. (B) Monitoring of daily maximum temperature, daily maximum HR, WBC, NE%, CRP, and PCT levels. CRP = C-reactive protein, HR = heart rate, NE% = percentage of neutrophil count, PCT = procalcitonin, Temp = temperature, WBC = white blood cell.

The patient underwent a distal pancreatectomy on July 10, 2024. On the day of pancreatectomy (day 0), he was started administering Cefoperazone/sulbactam 3 g q8h and Ornidazole 0.5 g q12h as antibacterial treatment for infection prevention, along with Omeprazole enteric-coated tablets 20 mg once a day to reduce stomach acid. Additionally, *Bacillus licheniformis* capsules 0.5 g were administered 3 times a day to regulate the intestinal flora. The patient’s condition remained stable, and on postoperative day 11, bifid triple-viable capsules 0.42 g twice a day to further support intestinal flora regulation. Both white blood cell (WBC count, 11.64 × 10^9/L) and C-reactive protein (CRP) (8.91 mg/L) levels began to rise, but the neutrophil percentage (NE%) remained on postoperative day 14. Unfortunately, on postoperative day 16, the patient developed chills, tachycardia with a heart rate of 132 bpm, tachypnea with a respiratory rate of 30 bpm, and decreased oxygen saturation to 91%. WBC and CRP levels continued to rise, and NE% also increased to 86%, with procalcitonin (PCT) reaching 13.6 ng/mL. Additionally, multiple patchy skin changes were noted in the trunk and limbs. Treatment included oxygen therapy via face mask, meropenem 1 g q8h, and vancomycin 1 g q12h for infection management, as well as dexamethasone 5 mg for anti-inflammatory treatment. Blood cultures were sent for testing and returned positive results in 4 bottles: *B. licheniformis* and *L. plantarum* in anaerobic bottles of peripherally inserted central catheter (PICC), *B. licheniformis* and *K. pneumoniae* in aerobic bottles of PICC, and *K. pneumoniae* in aerobic and anaerobic bottles of peripheral blood. On the same day, PICC culture of the patient revealed only *K. pneumoniae* (without *B. licheniformis* and *L. plantarum*). The temperature reached its highest point of 38.0°C, and the WBC count peaked at 26.32 × 10^9^/L on postoperative day 17, while both CRP and PCT reached their highest levels (191.54 mg/L and 30.80 ng/mL) respectively on postoperative day 18. The final treatment plan was adjusted to tigecycline 50 mg q12h and moxifloxacin 400 mg qd on postoperative Day 19. WBC, NE%, and CRP levels began to decrease, and PCT significantly dropped to 6.36 ng/mL. On postoperative days 23 and 29, the blood cultures were negative. The clinical course and therapeutic regimen used in this case are shown in Figure 1. Written informed consent was obtained from the family members of each patient. This study was approved by the research ethics board of Aerospace Center Hospital (permission no. 2024051).

## 3. WGS for homology analysis and pathogenicity prediction

To determine whether the *B. licheniformis* and *L. plantarum* detected in the patient’s blood cultures originated from oral probiotics, bacterial cultures were conducted on bifid triple-viable capsules and *Bacillus licheniformis* capsules obtained from the patient. The drug strains were identified using VITEK^®^ MS (BioMérieux, Lyon, France) as well as the bacteria isolated from blood cultures, confirming the presence of *B. licheniformis* and *L. plantarum*. Pure cultures obtained from the patient and probiotics were further analyzed using WGS at Shanghai Majorbio Bio-Pharm Technology Co., Ltd. Genomic DNA was extracted from the strains using a TIANamp Bacteria DNA Kit (TIANGEN, Beijing, China). DNA quality was assessed using a NanoDrop ND-1000 spectrophotometer (NanoDrop Technologies Inc., Wilmington) and gel electrophoresis. Illumina PE150 sequencing was performed after the library passed quality inspection. Raw data were trimmed and filtered using the NGSQCToolkit (v2.3), and duplicate reads were removed using FastUniq (v1.1). High-quality reads were further corrected using BLESS (v1.01). The genome assembly was performed using SOAPdenovo (v2.04) and GapCloser (v1.12). The assembled scaffolds were annotated using the following databases: NR Database (version 20230830), Swiss-Prot Database (version 202312), Pfam Database (version 36), eggNOG database (version 2020.06), GO Database (version 20230830), and KEGG Database (version 20230830). The genome scaffold characteristics of the 4 strains are presented in Table 1.

**Table 1:** General features of the genome scaffold of the 4 strains in this study.

Strain	Genome size (bp)	Scaffold no.	GC ratio (%)	CDS no.	tRNA no.	rRNA no.	16S rRNA no.	sRNA no.	Tandem repeat no.	SINE no.	LINE no.	LTR no.	DNA transposon no.
*B. licheniformis*_Drug	4452568	51	45.79	4520	80	6	0	99	94	7	31	0	3
*B. licheniformis*_Patient	4465173	49	45.79	4522	79	8	1	98	102	7	31	0	2
*L. plantarum*_Drug	3175310	23	44.63	2988	61	2	1	35	74	7	10	1	6
*L. plantarum*_Patient	3174584	24	44.63	2991	61	3	1	35	71	7	10	1	6

CDS = coding sequence, LINE = long interspersed nuclear element, LTR = long terminal repeated, SINE = short interspersed nuclear element.

**Figure 2.**
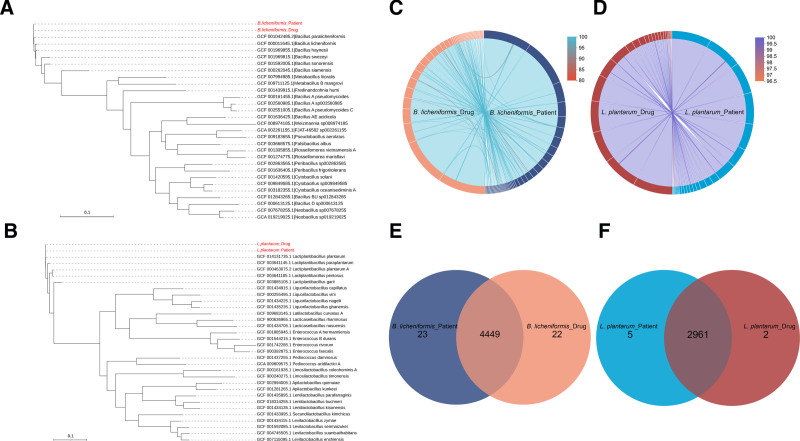
Whole-genome sequencing for homology analysis. (A) Phylogenetic tree of *B. licheniformis* from patient blood cultures and oral probiotics. (B) Phylogenetic tree of *L. plantarum* from patient blood cultures and oral probiotics. (C) Collinear blocks of *B. licheniformis* from patient blood cultures and oral probiotics. (D) Collinear blocks of *L. plantarum* isolated from patient blood cultures and oral probiotics. (E) Strain homologous genes of *B. licheniformis* from patient blood cultures and oral probiotics. (F) Strain homologous genes of *L. plantarum* isolated from patient blood cultures and oral probiotics.

**Figure 3.**
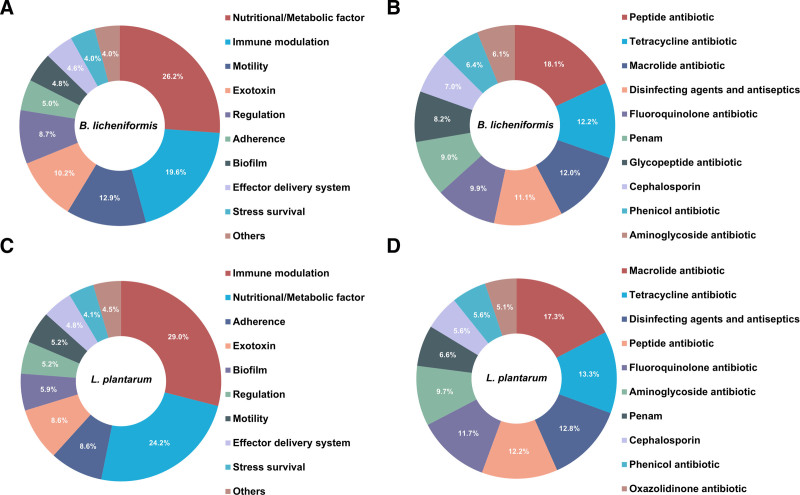
Whole-genome sequencing for pathogenicity prediction. (A) Virulence factor genes of *B. licheniformis*. (B) Antibiotic resistance genes of *B. licheniformis*. (C) Virulence factor genes of *L. plantarum*. (D) Antibiotic resistance genes of *L. plantarum*.

To analyze the homology of strains from the patient and oral probiotics, we constructed a phylogenetic tree of housekeeping genes using MEGA (v10.1.7) (Fig. 2A and B). Collinear blocks were analyzed using MUMmer (v3.23) (Fig. 2C and D). Homologous genes of the strains were also analyzed using OrthoMCL (version 14-137) (Fig. 2E and F). The results showed that the evolutionary distance and relatedness between the 2 *B. licheniformis* strains were similar to those between the 2 *L. plantarum* strains. The average nucleotide identity of *B. licheniformis* (99.99466%) and *L. plantarum* (99.99524%) was calculated using the Majorbio Cloud Platform (https://cloud.majorbio.com/page/tools).^[7]^ Multilocus sequence typing was performed using the PubMLST dataset, revealing that the 2 *B. licheniformis* strains corresponded to ST-49, whereas no ST typing comparison results were available for the 2 *L. plantarum* strains (Table 2). We concluded that the 2 gram-positive strains found in the patient’s blood cultures were homologous to those present in oral probiotics. Pathogenicity prediction based on WGS included virulent factors aligned with the VFDB Database (version 20240301) and antibiotic resistance aligned with the CARD Database (v3.2.9). The virulence factors of *B. licheniformis* (Fig. 3A) from both patients and probiotics were similar, encompassing nutritional/metabolic factors, immune modulation, motility, exotoxins, regulation, adherence, biofilm formation, effector delivery systems, and stress survival. The antibiotic resistance genes (Fig. 3B) in both *B. licheniformis* strains exhibited similarities, primarily showing resistance to peptides, tetracyclines, macrolides, and fluoroquinolones. Similarly, the virulence factors of *L. plantarum* (Fig. 3C) included immune modulation, nutritional/metabolic factors, adherence, exotoxins, biofilm formation, motility, regulation, effector delivery systems, and stress survival, along with gene resistance (Fig. 3D) to macrolides, tetracyclines, peptides, fluoroquinolones, and aminoglycosides.

**Table 2:** The MLST of *B. licheniformis* and *L. plantarum*.

Strain source	Genetic type	Housekeeping gene					
		spo0A	recF	ccpA	rpoB	sucC	adk
*B. licheniformis*_Drug	ST-49	8	5	7	3	3	3
*B. licheniformis*_Patient	ST-49	8	5	7	3	3	3
*L. plantarum*_Drug	ST-Unknown	–	–	–	–	–	–
*L. plantarum*_Patient	ST-Unknown	–	–	–	–	–	–

MLST = multilocus sequence typing.

## 4. Discussion

In this case, the patient developed severe clinical symptoms associated with a bloodstream infection, and blood cultures revealed *K. pneumoniae*, *B. licheniformis*, and *L. plantarum*. Based on previous experience, *K. pneumoniae* is typically considered to be the primary pathogen responsible for the observed clinical symptoms. However, the presence of *B. licheniformis* (2 bottles) and *L. plantarum* (1 bottle) raises concerns about their potential sources. After discussing the medication process with the surgeon-in-charge and conducting a homology analysis of the strains, we supposed that the 2 bacteria originated from *B. licheniformis* capsules and Bifid. Triple-viable capsules of oral administration showing over 99% patient-drug homology. We also performed surface cultures of the patients’ hands, clothing, and personal belongings to rule out possible sources of contamination, and the results showed no presence of *B. licheniformis* or *L. plantarum*. Environmental hygiene assessments of the patient’s ward and laboratory, where bacterial cultures were conducted, showed no presence of these 2 bacteria. Moreover, PICC culture only revealed *K. pneumoniae* without *B. licheniformis* and *L. plantarum*. Based on the above results, we are confident that *B. licheniformis* and *L. plantarum* in blood cultures originated from the oral probiotics entering the bloodstream. Ultimately, discontinuation of oral probiotics and timely antibiotic treatment resulted in a negative blood culture result.

The genera and species of probiotics usually include lactic acid bacteria, such as Lactobacillus, Streptococcus, Lactococcus, Pediococcus, and Enterococcus, as well as Bifidobacterium, Propionibacterium, Bacillus, and *Escherichia coli*. *B. licheniformis* can rapidly colonize the surfaces of intestinal mucosal epithelial cells, forming a membrane flora in conjunction with normal intestinal bacteria, such as *Lactiplantibacillus* spp. This process helps prevent the adhesion of pathogenic bacteria and promotes the effective proliferation of beneficial bacteria, thereby enhancing the immune barrier functions of the body.^[8]^ Additionally, *L. plantarum* produces bacteriocins with noteworthy antimicrobial activity.^[9]^ Supplementation with probiotics in individuals over the age of 69 years has been shown to not only increase the levels of potentially beneficial intestinal bacteria but also enhance the activation of the nonspecific immune response.^[10]^ The safety of probiotics is closely linked to their intended use, which involves considering factors such as the consumer or patient’s potential vulnerabilities, dosage and duration of consumption, and method and frequency of administration.^[10]^ The decision to supplement our patient with *B. licheniformis* and *L. plantarum* was based on irritable bowel syndrome and gut microbiome dysbiosis. As there are no established clinical practice guidelines outlining a specific adjustment plan for vulnerable patients, the treatment regimen and probiotic dosage in this case followed the drug instructions. Based on the patient’s medical history, there was no history of connective tissue disease, malignant tumor, or long-term use of immunosuppressive drugs. However, the patient’s advanced age and multiple chronic diseases contributed to a weakened immune system. Furthermore, surgery and pneumonia can result in frailty. Ultimately, a damaged intestinal barrier may provide a pathway for bacterial translocation into the bloodstream. Studies have indicated that critically ill patients who consume probiotics may face an increased risk of bacterial translocation.^[11]^ Currently, *B. licheniformis* has been reported of *B. licheniformis* enter the bloodstream or cause bacteremia and even sepsis. Patients in these cases all exhibited vulnerability factors, such as extremely low birth weight infants, hematological malignancies, undergoing major invasive surgeries, or had central venous catheters in place, as well as those with weakened immune function or long-term steroid use.^[12,13]^ However, no bacteremia caused by *L. plantarum* has been reported to date.

Probiotics are unique because they are live microorganisms when administered. Unlike other food and drug ingredients, they have the potential to produce toxins or harmful metabolites in situ. *Klebsiella pneumoniae*, a confirmed bloodstream infection pathogen, caused severe clinical symptoms in our study, and effective antibiotic treatment quickly cleared the blood culture. There was no clear evidence that *B. licheniformis* and *L. plantarum* caused severe infections in this case; thus, we used WGS to predict the virulence factors of the 2 strains. The results identified several virulence factors, including immune modulation, nutritional/metabolic factors, adherence, exotoxins, biofilm formation, motility, regulatory elements, effector delivery systems, and stress-survival mechanisms. These factors are closely associated with clinical infection. Immune-modulating factors suppress the host immune response, causing chronic infections or excessive inflammation. Adhesion factors enable bacterial attachment to host cells or surfaces, leading to colonization and invasion. Exotoxins (toxic proteins) damage host cells or disrupt their functions. Biofilm formation protects bacteria, helps them resist immunity and antibiotics, and causes chronic or medical device-related infections. Research indicates that *B. licheniformis* can produce a glutamate polymer that forms a biofilm, contributing to persistent and recurrent bacteremia.^[14]^ Additionally, *Bacillus* spp. strains are known to produce various toxins, including hemolysins and enterotoxins.^[2]^ These toxins have been validated through cell cytotoxicity assays, and severe cases of food poisoning have been documented.^[15]^ Tleyjeh et al.^[16]^ reported the first case of Fournier gangrene affecting the scrotum and penile tissues caused by *Lactiplantibacillus* spp. Another issue is the bacterial resistance. However, there is a lack of literature regarding antibiogram tests for *L. plantarum*, we can only reference the antibiotic resistance results inferred from WGS, which include resistance to macrolides, tetracyclines, peptides, and fluoroquinolones, among others. The presence of transferable antibiotic resistance genes through plasmids or conjugative transposons poses a theoretical risk of transfer to a more harmful member of the gut microbiota. *B. licheniformis* is generally susceptible to carbapenems, glycopeptides, aminoglycosides, quinolones, chloramphenicol, peptolides, and fusidic acid but resistant to penicillin, fosfomycin, macrolides, and nitroimidazoles,^[5,12,17]^ which is roughly similar to our WGS prediction results. We believe that after excluding the possibility of contamination, the detection of *B. licheniformis* and *L. plantarum* in the patient’s blood culture, regardless of whether they cause severe clinical symptoms, makes the utilization of Vancomycin and Moxifloxacin a prudent approach.

The benefits of probiotics in maintaining the gut microbiota balance, enhancing immunity, and alleviating inflammation should not be overlooked. Recent advances in microbiome and probiogenomics have made probiotics not only a health supplement but also a potential aid in the treatment of multiple diseases.^[18,19]^ Numerous companies are now developing genetically modified microorganisms and microbial agents derived from the stool of healthy donors, which are classified as biotherapeutic products for microbial therapeutics.^[18]^ However, no probiotic products currently available in the market can claim to diagnose, treat, or cure diseases. This is because of the lack of rigorous clinical data on their effectiveness under pathological conditions. Therefore, many conditionally approved clinical trials, similar to those for drugs and medical devices, need to be conducted. Clinicians and pharmacists must increase awareness of the safety of probiotics, particularly for at-risk populations, and hospital systems should remain vigilant for potential rare adverse events.^[20]^ Authorities should prioritize probiotic regulations, establish screening standards, strengthen oversight, combat false advertising, and mandate safety clinical studies. Our study acknowledged single-case reports with limitations in determining the safety and risks of probiotic use. More similar cases should be observed in clinical practice.

## 5. Conclusion

Probiotics are generally considered relatively safe, but should be preceded by risk screening in vulnerable populations. WGS revealed the potential risks of probiotic use through homology analysis and prediction of virulence factors and antibiotic resistance.

## Acknowledgments

We are thankful to Dr Fangze Li and Shanghai Majorbio Bio-Pharm Technology Co., Ltd. for their excellent technical assistance.

## Author contributions

**Conceptualization:** Tianqi Qi, Wenqiang Li.

**Data curation:** Tianqi Qi, Yingshi Wang, Yanhui Liu, Wenqiang Li.

**Formal analysis:** Tianqi Qi, Yingshi Wang, Yanhui Liu.

**Funding acquisition:** Tianqi Qi.

**Investigation:** Wenqiang Li, Shanshan Wu.

**Methodology:** Shanshan Wu.

**Project administration:** Wenqiang Li.

**Supervision:** Wenqiang Li.

**Validation:** Tianqi Qi, Yanhui Liu, Wenqiang Li.

**Visualization:** Tianqi Qi, Yingshi Wang, Yanhui Liu.

**Writing – original draft:** Tianqi Qi.

**Writing – review & editing:** Shanshan Wu.
